# Identification and tissue distribution of chemosensory protein and odorant binding protein genes in *Tropidothorax elegans* Distant (Hemiptera: Lygaeidae)

**DOI:** 10.1038/s41598-018-26137-6

**Published:** 2018-05-17

**Authors:** Yue-Qin Song, Hui-Zhong Sun, Jun Du

**Affiliations:** 1 Forestry College, Henan University of Science and Technology, Luoyang, 471000 China; 2 Institute of Plant Nutrition and Resource Environment, Henan Academy of Agricultural Sciences, Zhengzhou, 450002 China

## Abstract

*Tropidothorax elegans* Distant (Hemiptera: Lygaeidae) is an insect pest that inflicts damage to vegetables and flowering plants across China. The olfactory system regulates insect behavior, such as feeding, mating, oviposition and predator avoidance. Odorant-binding proteins (OBPs) and the chemosensory proteins (CSPs) are two groups of small soluble proteins that initiate olfactory signal transduction in insects. In this study, we generated antennal transcriptomes of male and female *T*. *elegans*, and identified 19 putative OBP (14 classic OBPs and five plus-C OBPs) and seven CSP genes. Through real-time quantitative PCR analysis, we found that 14 of the 19 OBP genes were highly expressed in the antennae of both adult females and males, and 3 OBP genes were expressed higher in the antennae of males than females. Some OBP genes were also highly expressed in the legs or wings. Three CSP genes were highly expressed in the antennae of both sexes, and *TeleCSP7* showed higher expression in male antennae compare to females. Interestingly, one CSP gene, *TeleCSP2*, was expressed in all olfactory tissues. Our results provide molecular insights into further investigating of the olfactory system of an important plant pest, *T*. *elegans*.

## Introduction

The olfactory system plays an important role in regulating behaviors in insects, such as finding food, mating, oviposition and avoiding predators^1,2^. The process of olfactory perception is mediated by a series of proteins that include odorant-binding proteins (OBPs), chemosensory proteins (CSPs), olfactory receptors (ORs), sensory neuron membrane proteins (SNMPs) and olfactory degrading enzymes (ODEs). OBPs and CSPs are produced in the lymph of the chemosensilla and bind to chemical signals from the external environment, then transfer odorants through the sensillar lymph to the ORs, activating signal transduction^2,3^. Therefore OBPs and CSPs are two families of small water-soluble polypeptides in the lymph of chemosensilla and initiate the biochemical recognition of olfactory signals in insect^4–6^.

All insect OBPs have six highly conversed cysteine residues, paired in three interlocked disulphide bridges^7^. OBPs are classified into five groups: ‘Classic’ OBPs with six Cys residues, ‘dimer’ OBPs with two classical Cys signature motifs, ‘Minus-C’ OBPs that lacks two of the six conserved Cys, ‘Plus-C’ OBPs that has a Pro residue and two additional Cys residues and ‘Atypical’ OBPs with 9–10 Cys residues^5^. OBPs are highly expressed in the antennae, and the primary function of OBPs are to transport odorant molecules to ORs in the antenna^8,9^. However, recent studies have found expression of OBPs in nonsensory organs, such as pheromone glands and reproductive organs. This suggests that OBPs serve an additional function as chemical compound carrier, which may be analogous to the vertebrates urinary and salivary proteins as well as some insect chemosensory proteins^10,11^.

In contrast to OBPs, CSPs have four conserved Cys residues^5,12^. Initially, these proteins were named as olfactory specific protein D (OS-D)^13^ or A10^14^, but were then named chemosensory proteins (CSPs). CSPs are expressed in chemosensory tissues, such as antennae^15^, maxillary palps^16^, proboscis^17^, labial palps^16,18^, wings^19^, legs^20^, and non-chemosensory tissues, including pheromone gland^21,22^ and ejaculatory bulb^23^. CSPs may play important role as carriers for odorant molecules through the sensillar lymph to transmembrane chemoreceptors but can also be involved in other physiological and behavioral functions.

The number of OBPs and CSPs is highly variable even between closely related species. For example, 38 OBPs were identified in *Apolygus lucorum* Meyer-Dür^9^, 33 OBPs in *Lygus lineolaris* Palisot de Beauvois^8^, 28 OBPs and 16 CSPs in *Nysius ericae* Schilling^24^, 16 OBPs and 8 CSPs in *Adelphocoris suturalis* Jakovlev^25^, 14 OBPs and 3 CSPs in *Adelphocoris lineolatus* Goeze^26,27^, 10 OBPs and 5 CSPs in *Cyrtorhinus lividipennis* Reuter^28^, 40 OBPs and 11 CSPs in *Empoasca onukii* Matsuda^29^, 8 OBPs and 13 CSPs in *Bemisia tabaci* Gennadius^30^, 30 OBPs in *Halyomorpha halys* Stål^31^, 9 OBPs and 3 CSPs in *Pachypeltis micranthus* Mu et Liu^32^.

*Tropidothorax elegans* Distant (Hemiptera: Lygaeidae) is a polyphagous insect pest that can severely damage commercially important plants, such as locust, pepper, cucurbit, red sage, oilseed rape, Chinese cabbage, and wheat^33^. Insects detect the chemical information of a plant to determine whether it is a suitable host. However, the molecular mechanisms underlying the detection of host plants remain elusive. In this study, we identified OBP and CSP genes from *T*. *elegans* by screening the antennal transcriptome and subsequently examined their expression in different tissues by real-time quantitative PCR (RT-qPCR). Our results provided resources for further functional characterization of *T*. *elegans* OBPs/CSPs.

## Results

### Identification of CSP and OBP genes in *T*. *elegans*

After removing adaptors and low quality reads, a total of 36,445,539 clean reads were obtained with a Q30 percentage of 91.90%. The assembly of all clean reads together led to the generation of 118,998 (mean length 852.84 bp) transcripts and 93,940 (mean length 667.34 bp) unigenes with N50 length of 1,545 and 1,046 bp. We obtained 33,079 unigenes that were longer than 500 bp, which accounted for 35.21% of all unigenes (Table S1).

The tBLASTn results showed 19 unigenes encoding putative OBP genes (GenBank Accession Nos. MF593898–MF593916). Of the 19 TeleOBPs that were identified, 17 TeleOBPs had intact ORFs that were 140–383 aa in length, and 15 TeleOBPs had a signal peptide at the N-terminus. Two TeleOBPs (TeleOBP2 and TeleOBP6) were partial sequences with the N-terminus missing. The full-length protein sequences of TeleOBP1–19 had molecular weights of 15.64–23.38 kDa and isoelectric points of 4.27–8.86. BLAST analyses revealed that the TeleOBP sequences were similar to putative OBP sequences identified from other hemipteran species. The highest degree of sequence identity (75%) was found in the putative OBP from the alfalfa plant bug species, *A*. *lineolatus*, followed by *A*. *suturalis* (53%), *L*. *lineolaris* (53%) and *Telenomus podisi* (53%) (Table 1).

**Table 1:** Sequence information of TeleOBPs and TeleCSPs.

Gene name	Acc. no.	ORF(aa)	Molecular weight (kDa)	Isoelectric point	Signal peptide	Full length	Best Blastx hit					
							Gene annotation	Species	Protein ID	Score(bits)	E-volue	identify
TeleOBP1	MF593898	205	22.66	5.18	1–21	yes	OBP7	Adelphocoris suturalis	ANA10233.1	191	3e-58	53%
TeleOBP2	MF593899	102	11.41	5.16	no	No	OBP5	Adelphocoris lineolatus	ACZ58031.1	164	4e-50	75%
TeleOBP3	MF593900	152	17.04	4.27	1–29	yes	OBP3	Euschistus heros	AIU64820.1	85.8	3e-18	36%
TeleOBP4	MF593901	140	15.85	5.43	1–17	yes	OBP4	Chinavia ubica	AIU64827.1	100	3e-24	39%
TeleOBP5	MF593902	383	45.43	7.96	1–23	yes	OBP5	Halyomorpha halys	AOV87022.1	209	2e-61	40%
TeleOBP6	MF593903	156	17.09	8.86	1–13	no	OBP25	Chilo suppressalis	ANC68513.1	56.6	7e-07	29%
TeleOBP7	MF593904	200	22.96	5.4	1–20	yes	OBP3	Sogatella furcifera	AGZ04903.1	64.3	3e-09	32%
TeleOBP8	MF593905	203	22.83	6.34	1–20	yes	OBP31	Apolygus lucorum	AMQ76484.1	104	2e-24	34%
TeleOBP9	MF593906	172	19.11	7.56	no	yes	OBP1	Pachypeltis micranthus	ARO46430.1	82	9e-17	33%
TeleOBP10	MF593907	143	15.64	5.33	1–18	yes	OBP2	Chinavia ubica	AIU64825.1	110	2e-28	42%
TeleOBP11	MF593908	155	17.69	5.26	1–19	yes	OBP4	Chinavia ubica	AIU64827.1	63.2	1e-09	32%
TeleOBP12	MF593909	111	12.56	9.18	no	yes	OBP33	Apolygus lucorum	AMQ76486.1	93.6	4e-22	45%
TeleOBP13	MF593910	175	19.69	4.84	1–17	yes	OBP13	Halyomorpha halys	AOV87030.1	63.5	2e-09	33%
TeleOBP14	MF593911	152	17.07	5.57	1–20	yes	OBP2	Telenomus podisi	AIU64830.1	166	4e-50	53%
TeleOBP15	MF593912	145	16.04	6.31	1–18	yes	OBP15	Halyomorpha halys	AOV87032.1	98.2	2e-23	31%
TeleOBP16	MF593913	152	17.04	5.16	1–19	yes	OBP27	Halyomorpha halys	AOV87044.1	103	2e-25	35%
TeleOBP17	MF593914	140	15.78	4.97	1–22	yes	OBP4	Lygus lineolaris	AHF71031.1	162	4e-49	53%
TeleOBP18	MF593915	262	30.92	7.05	1–20	yes	OBP24	Anopheles funestus	ADQ01710.1	53.1	4e-05	31%
TeleOBP19	MF593916	210	23.38	6.32	1–25	yes	OBP25	Apolygus lucorum	AMQ76478.1	122	2e-31	34%
TeleCSP1	MF598723	134	15.29	8.93	1–21	yes	CSP8	Nilaparvata lugens	ACJ64054.1	149	9e-44	57%
TeleCSP2	MF598724	115	12.88	9.27	1–26	no	CSP13	Lygus hesperus	APB88051.1	96.3	1e-23	42%
TeleCSP3	MF598725	125	14.06	5.59	1–19	yes	CSP3	Apolygus lucorum	AEP95757.1	152	3e-45	58%
TeleCSP4	MF598726	141	16.56	6.41	1–23	yes	CSP4	Adelphocoris suturalis	ANA10246.1	124	3e-34	47%
TeleCSP5	MF598727	127	14.47	4.70	1–17	yes	CSP8	Lygus hesperus	APB88044.1	130	1e-36	48%
TeleCSP6	MF598728	123	13.98	9.18	1–17	yes	CSP3	Apolygus lucorum	AGD80083.1	209	8e-68	86%
TeleCSP7	MF598729	135	15.66	5.74	1–18	yes	CSP8	Lygus hesperus	APB88044.1	119	2e-32	46%

**Figure 1:**
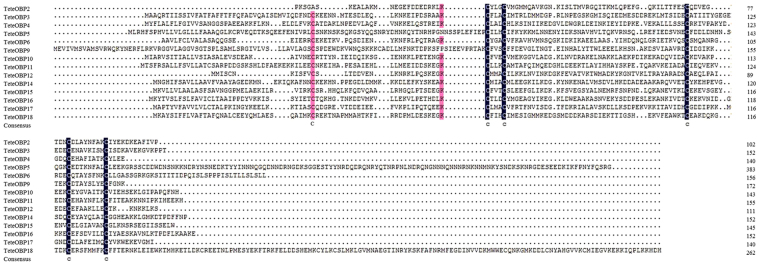
Alignment of *Tropidothorax elegans* ‘classic’ odorant-binding proteins (OBPs). The six conserved cysteine residues are indicated with the letter “c” under the sequence.

**Figure 2:**
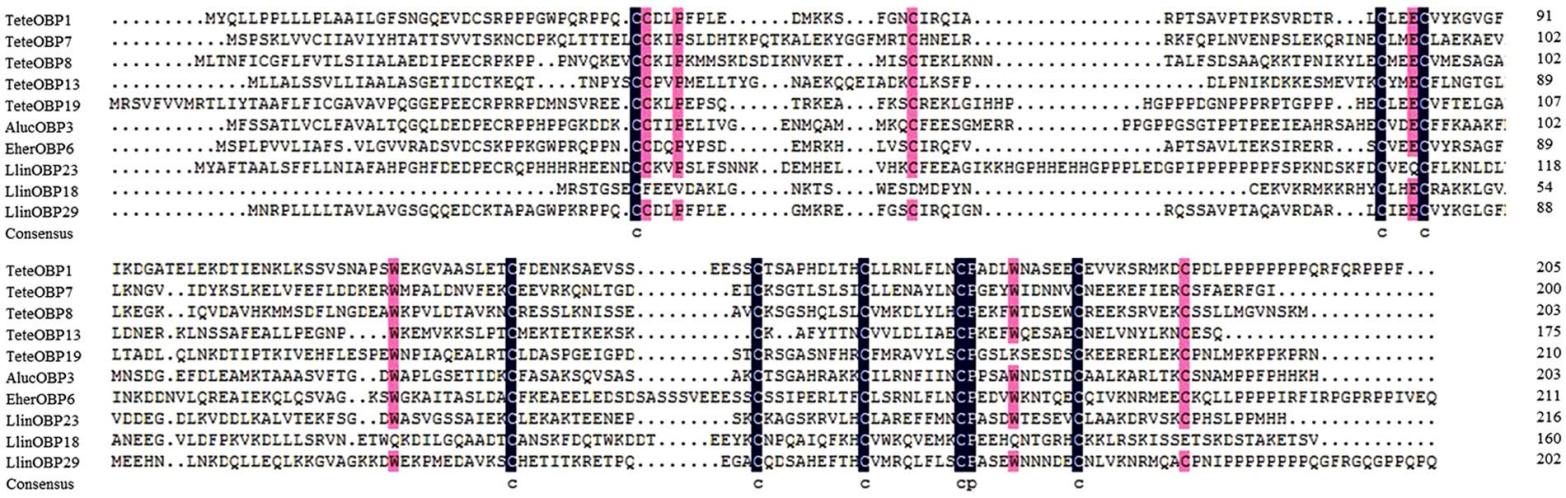
Alignment of *Tropidothorax elegans* ‘Plus-C’ odorant-binding proteins (OBPs). The conserved residues in the ‘Plus-C’ OBPs motif are indicated with the letter “c” under the sequence.

Except for TeleOBP2 where the 5′ information was missing, the other 18 TeleOBPs contained OBP cysteine patterns that were ‘classic’ OBP or ‘Plus-C’ OBP. Based on the Hemiptera ‘classic’ OBP Cys motif (C1-X_15–39_-C2-X_3_-C3-X_21–44_-C4-X_7–12_-C5-X_8_-C6), we classified 13 TeleOBPs (TeleOBP3–6, TeleOBP9–12 and TeleOBP14–18) as ‘classic’ OBPs (Fig. 1). The remaining five TeleOBPs (TeleOBP1, TeleOBP7–8, TeleOBP13 and TeleOBP19) were categorized as ‘Plus-C’ OBPs, because they contained two additional conserved cysteins and a conserved proline immediately after the sixth cysteine (Fig. 2).

We next searched for CSP genes and identified seven putative TeleCSP genes from the *T*. *elegans* antennal transcriptome. Aside from TeleCSP2, six TeleCSPs contained complete ORF ranging 115–141 aa. The sequences were named TeleCSP1 to TeleCSP7 (GenBank accession numbers MF585723–MF585729). The full-length protein sequences of TeleCSP1 to TeleCSP7 had molecular weights of 13.96–16.56 kDa and isoelectric points of 4.70–9.18. All TeleCSPs shared high sequence similarities to known hemipteran CSPs, and in particular, TeleCSP6 showed highest identity (86%) with a CSP from *A*. *lucorum* (Table 1).

**Figure 3:**
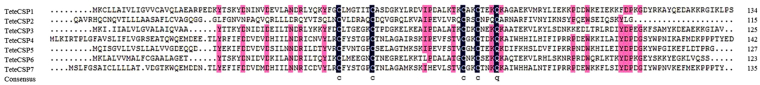
Alignment of *Tropidothorax elegans* chemosensory proteins (CSPs). Four conserved cysteine residues are indicated with the letter “c” under the sequence.

All TeleCSP genes contained a 17–26 aa N-terminal signal peptide sequence and a highly conserved four cysteine residues with the spacing pattern C_1_-X_6_-C_2_-X_18_-C_3_-X_2_-C_4_ (Fig. 3).

### Phylogenetic analysis

**Figure 4:**
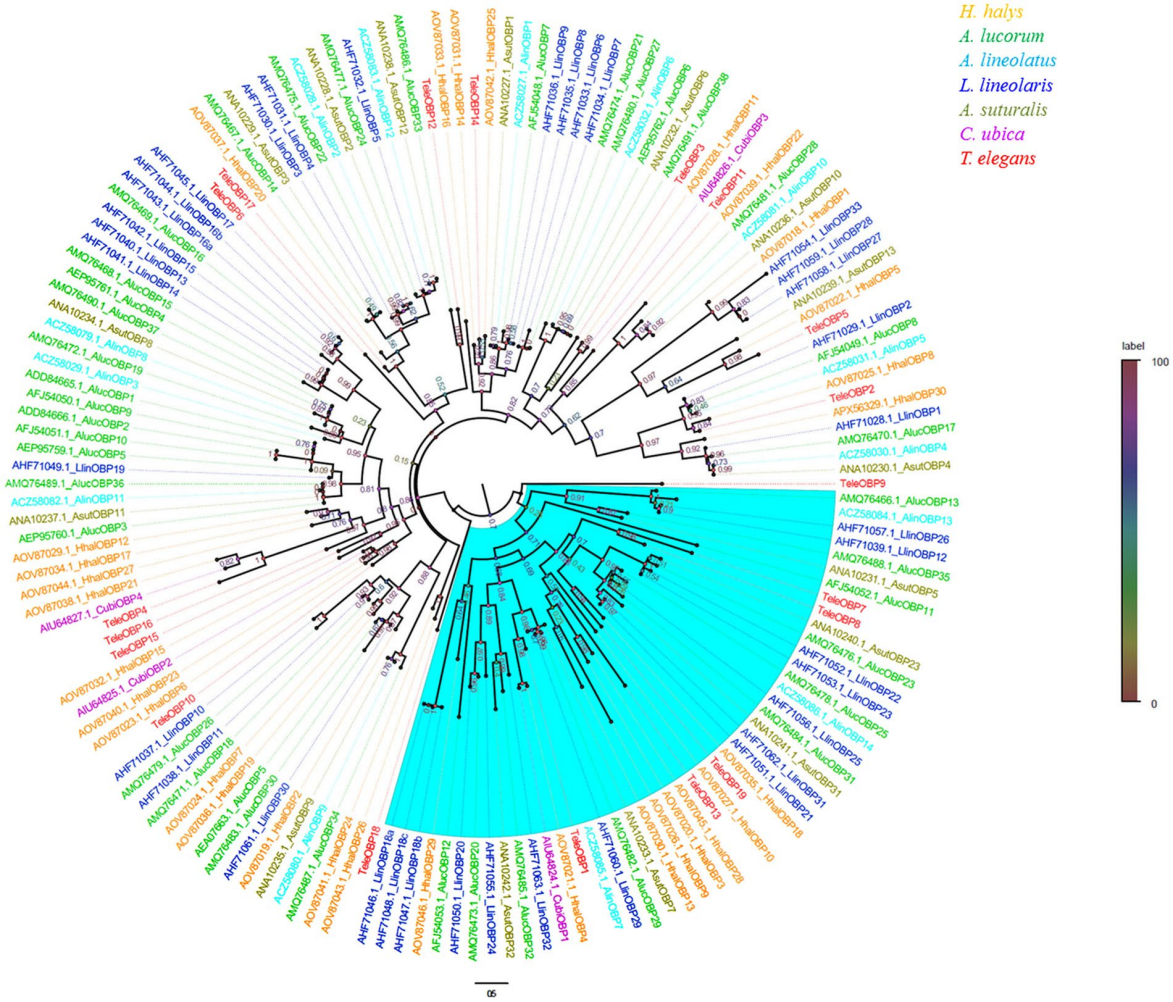
Phylogenetic relationships of 160 odorant-binding proteins (OBPs) from seven Hemiptera species: *Halyomorpha halys, Apolygus lucorum, Adelphocoris lineolatus, Lygus lineolaris, Adelphocoris suturalis, Chinavia ubica*, and *Tropidothorax elegans*. The branch leading to ‘Plus-C’ odorant-binding proteins (OBPs) is marked in blue.

To conduct a phylogenetic analysis of OBPs, we used a total of 160 OBP protein sequences from seven different Hemiptera insects, including the 19 predicted OBPs we identified from *T*. *elegans*, 30 OBPs from *H*. *halys*, 39 OBPs from *A*. *lucorum*, 14 OBPs from *A*. *lineolatus*, 39 OBPs from *L*. *lineolaris*, 15 OBPs from *A*. *suturalis*, and four OBPs from *Chinavia ubica*. The resulting phylogenetic tree showed that OBPs were segregated into two clades: ‘classic’ OBP and ‘Plus-C’ OBPs. OBPs from the same species but in different OBP family (classic vs Plus-C) were equally divergent from each other. Most *T*. *elegans* OBPs were located in the same branch along with the orthologous sequences. However TeleOBP9 and TeleOBP18 had no orthologous sequences and segregated into unique clades (Fig. 4).

**Figure 5:**
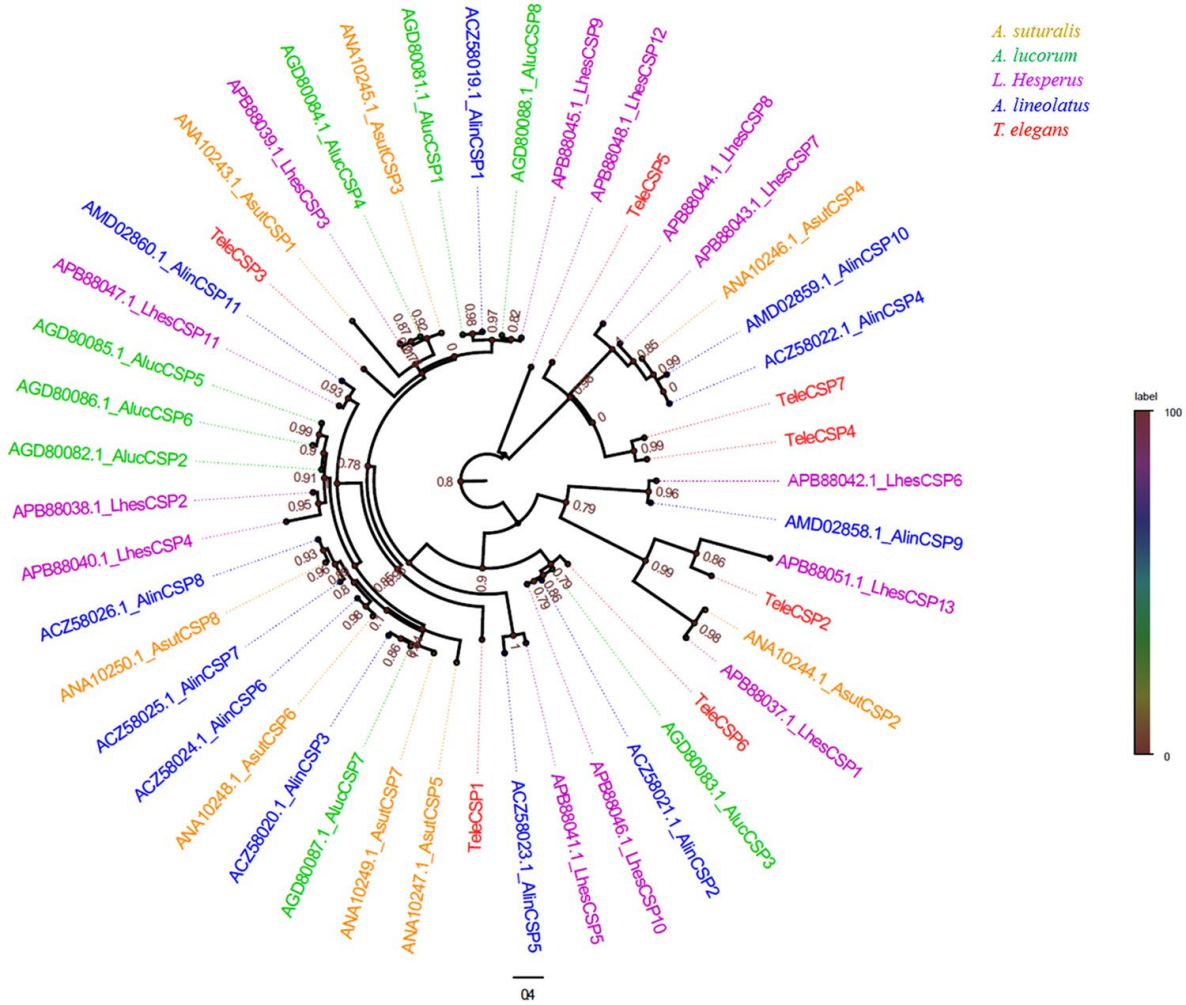
Phylogenetic relationships of 47 chemosensory proteins (CSPs) from five hemipteran species: *Adelphocoris suturalis*, *Apolygus lucorum*, *Lygus Hesperus*, *Adelphocoris lineolatus*, *Tropidothorax elegans*.

We next constructed a phylogenetic tree of CSP protein sequences using 47 CSP sequences from five hemipteran species, including seven CSPs from *T*. *elegans*, eight CSPs from *A*. *suturalis*, eight CSPs from *A*. *lucorum*, 13 CSPs from *Lygus hesperus*, and 11 CSPs from *A*. *lineolatus*. CSPs from the same species were also equally divergent from each other (Fig. 5).

### Tissue-specific expression of *T*. *elegans* OBP and CSP genes

**Figure 6:**
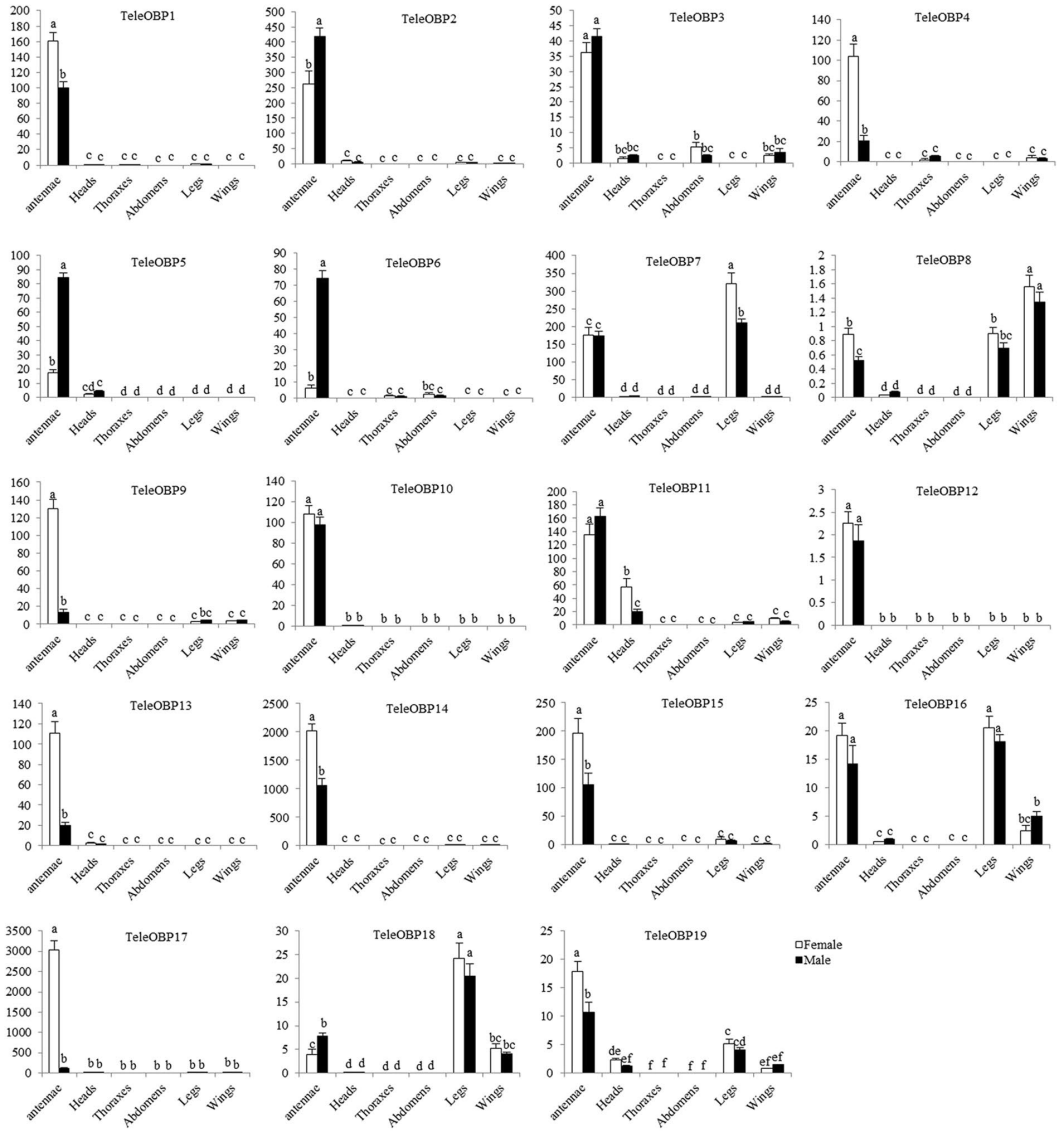
Relative expression of TeleOBP genes in female and male adult tissues of *Tropidothorax elegans* determined by RT-qPCR. TeleActin gene was used as an internal control. Data was normalized to actin expression levels and shown relative to the expression of female thoraxes each gene. Error bars represent standard errors of the means for three biological replicates and different lower cases letters indicate significant differences (*p* < 0.05).

To understand the function of TeleOBPs, we measured relative expression levels of OBP genes in different tissues of *T*. *elegans* by RT-qPCR. The antennal transcript levels of 14 TeleOBP genes (*TeleOBP1*, *TeleOBP2*, *TeleOBP3*, *TeleOBP4*, *TeleOBP5*, *TeleOBP6*, *TeleOBP9*, *TeleOBP10*, *TeleOBP11*, *TeleOBP12*, *TeleOBP13*, *TeleOBP14*, *TeleOBP15* and *TeleOBP19*) were significantly higher in both female and male antennae compared to other tissues. Furthermore, the expression levels of *TeleOBP2*, *TeleOBP5* and *TeleOBP6* were significantly higher in the antennae of males than the antennae of females. The expression levels of *TeleOBP1*, *TeleOBP4*, *TeleOBP9*, *TeleOBP13*, *TeleOBP14*, *TeleOBP15* and *TeleOBP17* were significantly higher in the female antennae than the male antennae. Interestingly, *TeleOBP7*, *TeleOBP16* and *TeleOBP18* were highly expressed in the legs. In addition, the Plus-C OBP *TeleOBP8* had highest expression levels in the wings of female and male compared to other tissues (Fig. 6).

**Figure 7:**
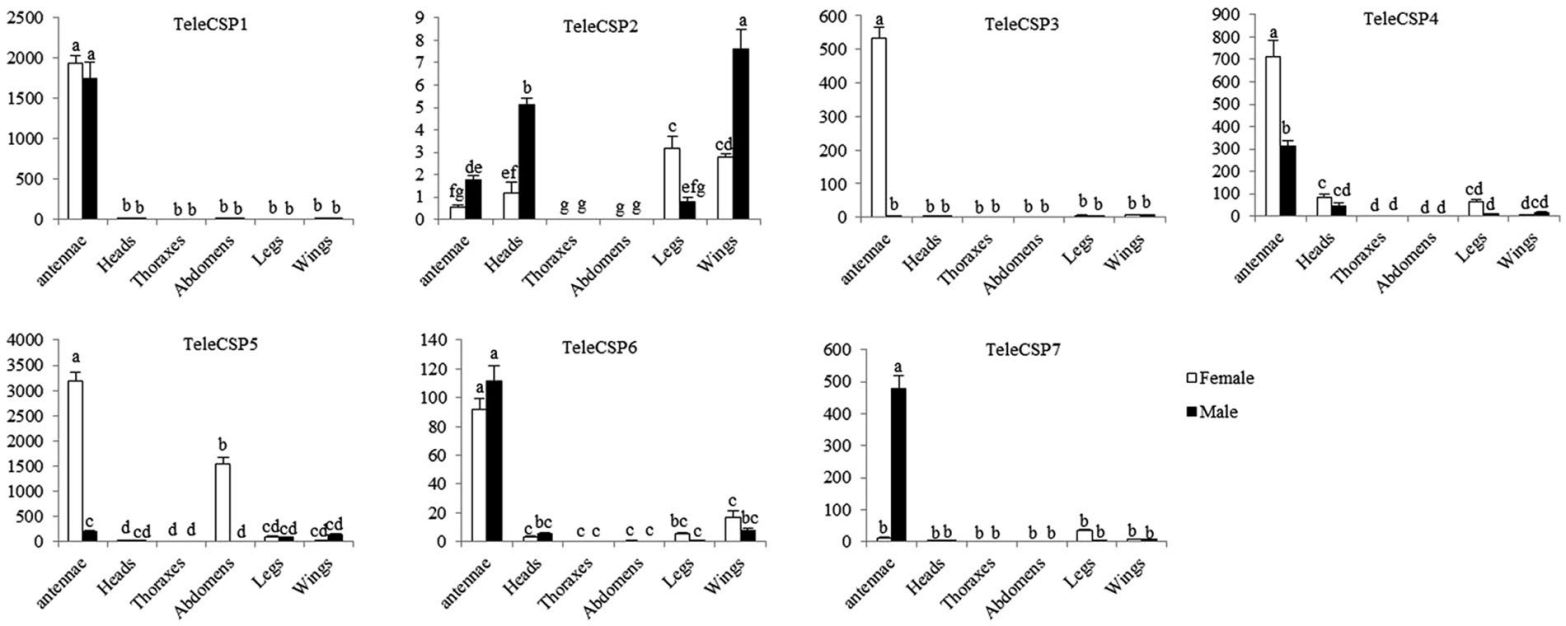
Relative expression of TeleCSP genes in female and male adult tissues of *Tropidothorax elegans* determined by RT-qPCR. TeleActin gene was used as an intermal control. Data was normalized to actin expression levels and shown relative to the expression of female thoraxes each gene. Error bars represent standard errors of the means for three biological replicates and different lower cases letters indicate significant differences (*p* < 0.05).

Of the seven TeleCSP genes, we found that *TeleCSP1*, *TeleCSP4* and *TeleCSP6* expression was highest in the antennae of females and males. *TeleCSP3*, *TeleCSP4* and *TeleCSP5* expression levels were significantly higher in the female antennae than the male antennae, while *TeleCSP1* and *TeleCSP6* expression levels were similar in females and in males. Interestingly, *TeleCSP2* was expressed in all olfactory tissues that were analyzed, which were antennae, head, legs and wings (Fig. 7).

## Discussion

Here, we identified 19 candidate OBP genes and seven CSP genes by searching the *T*. *elegans* antennal transcriptome. Although the number of OBP genes we identified in *T*. *elegans* is lower than other spcies, such as 38 OBPs in *A*. *lucorum*^9^, 33 OBPs in *L*. *lineolaris*^8^ and 28 OBPs in *N*. *ericae*^24^, but was similar to some Hemiptera species, such as 16 OBPs in *A*. *suturalis*^25^ and 14 OBPs in *A*. *lineolatus*^26^. The host range of *T*. *elegans* is broad, and there may be more OBP and CSP genes to detect odor molecules. Our analysis would not detect OBPs and CSPs primarily expressed in other tissues, or expressed at very low levels in the antennae, therefore the repertoire of TeleOBPs and TeleCSPs we identified may be incomplete.

The number of ‘Plus-C’ OBPs is variable even within the same order. For example, in five Hemiptera insects *L*. *lineolaris*, *N*. *ericae*, *A*. *lucorum*, *A*. *suturalis* and *A*. *lineolatus*, the number of ‘Plus-C’ OBPs is 10, 7, 6, 4 and 2, respectively^8,9,24–26^. Here, we identified five ‘Plus-C’ OBPs in *T*. *elegans*. The phylogenetic tree of Hemiptera OBPs revealed that except for TeleOBP9, all OBPs of *T*. *elegans* were assigned to orthologous Hemiptera OBPs clades. The ‘classic’ and ‘Plus-C’ OBP genes were clearly clustered in two clades, which may be due to the functional divergence of ‘classic’ OBP and ‘Plus-C’ OBP genes during evolution.

Expression patterns of OBPs can better help us to understand the function of these proteins. In Hemipterans, OBP genes are primarily expressed in the antenna. For example, 13 of 16 OBPs in *A*. *suturalis*^[25]^, 12 of 14 OBPs in *A*. *lineolatus*^[26]^, at least 6 of 10 OBPs in *Nilaparvata lugens*^34^, and 21 of 33 OBPs in *L*. *lineolaris*^[8]^ are expressed in the antennae. We found that 15 of 19 OBPs in *T*. *elegans* showed highest expression levels in the antennae, indicating a vital olfactory role of OBPs. Furthermore, eight TeleOBPs showed female-biased expression and three TeleOBPs showed male-biased expression. The occurrence of sex-biased expression suggests that these OBP genes may be involved in recognition of plant volatiles for oviposition behavior or pheromones^35–37^. OBPs expressed in taste sensilla on legs regulate behavioral adaptation to the host plant in *Drosophila sechellia*^38,39^. *TeleOBP7*, *TeleOBP16* and *TeleOBP18* were highly expressed in the legs, which may be related to host plant adaptation of *T*. *elegans*. Interestingly, the Plus-C OBP *TeleOBP8* was expressed higher in the wings of females and male than other tissues. OBP expression in non-olfactory tissues, which have been observed in a variety of species, suggests that they also may function as carriers of chemicals during developmental and physiological processes^9^.

In general, there are fewer CSP genes in Hemiptera insects than OBP genes. For example, there are 28 OBPs and 16 CSPs in *N*. *ericae*^24^, 16 OBPs and 8 CSPs in *A*. *suturalis*^25^, 14 OBPs and 3 CSPs in *A*. *lineolatus*^26^, 10 OBPs and 5 CSPs in *C*. *lividipennis*^28^, 40 OBPs and 11 CSPs in *E*. *onukii*^29^, and 9 OBPs and 3 CSPs in *P*. *micranthus*^32^. We identified seven CSPs in the antennal transcriptome of *T*. *elegans*. In our phylogenetic analysis, we found that there is low amino acid sequence conservation of CSPs among true bugs. Only one of seven TeleCSPs, TeleCSP6, clustered with AlucCSP3 with over 80% amino acid sequence identity. The distribution of CSP orthologs in other hemipterans suggests that CSP genes originating from a common ancestor may have similar functions or that they acquired novel functions via subfunctionalization^9^. These results suggest that CSP proteins in hemipteran insects undergo extensive gene duplication and divergence by natural selection, strongly indicating that they may have diverse functions^40^.

CSPs play an important role in a variety of biological process, including chemosensation^41^, leg regeneration^42,43^, and embryonic development^44^. *TeleCSP1*, *TeleCSP4* and *TeleCSP6* were expressed highly in the antennae, and maybe involved in recognizing sex pheromones and plant volatiles^27,45,46^. *TeleCSP2* was widely expressed in chemosensory and non-chemosensory tissues (antennae, head, legs and wings) and may have different physiological functions in adult tissues.

Overall, we generated the transcriptome of the female and male antenna of *T*. *elegans* by next-generatioan high throughput sequencing, and identified 19 OBP and seven CSP genes. Furthermore, we identified the gene expression patterns of CSPs and OBPs in different adult tissues. These findings provide important insights into the function of OBP and CSPs, and their role in odorant reception.

## Materials and Methods

### Insects samples and RNA extraction

A laboratory strain of *T*. *elegans* was generated from a population collected on a vegetable field in Luoyang, Henan, China (112-26′E, 34–43′N) in 2014. The population was reared on *Metaplexis japonica* (Thunb.) in a greenhouse maintained at 25 ± 2 °C, 14 h: 10 h light/dark cycle with 60~80% relative humidity. For the transcriptome, about 500 pairs of 3–4 days old female and male adult antennae were dissected, immediately frozen in liquid nitrogen, and stored at −80 °C until RNA isolation.

Total RNA was extracted using the RNAiso Plus kit (TaKaRa, Dalian, China) and treated with RNase-free DNase I (TaKaRa, Dalian, China) to remove residual DNA. The quantity and integrity of RNA was tested using 1.0% agarose gel electrophoresis and NanoDrop 2000c spectrophotometer (Thermo Scientific, USA), Qubit 2.0 (Life Technologies, USA) and Agilent 2100 (Agilent, USA).

### Antennal cDNA library construction, sequencing and analysis

Following the TruSeq RNA Sample Preparation Guide v2 (Illumina), mRNA was enriched using magnetic beads crosslinked to Oligo (dT), and fragmented into small pieces using the fragmentation buffer. First-strand cDNA was synthesized using small mRNA fragments with random primers and reverse transcriptase, and second-strand cDNA synthesis was conducted by adding dNTPs, DNA polymerase I and RNase H. Next, double stranded cDNA was purified with AMPure XP beads (Beckman Coulter, USA), and treated for end-repairing, Poly-A tailing and sequencing adapters linking. The size of the fragment was chosen using AMPure XP beads and the cDNA library was constructed by PCR amplification (Veriti^®^ 96-Well Thermal Cycle, Applied Biosystems, USA). The concentration and insert size of the cDNA library were detected using Qubit 2.0 and Agilent 2100, and quantified with q-PCR (CFX-96, Bio-Rad, USA).

Sequencing was performed by Genomics Services Lab of the Beijing Novogene Technologies Co., Ltd. (Beijing, China) using the Illumina HiSeq^TM^ 4000 platform to generate 150 bp pair-end reads. The raw data processing and base calling were performed using the Illumina instrument software.

Homologous sequences were searched using BLASTx and BLASTn against the Nr (non-redundant protein database) and Nt (non-redundant nucleotide sequence database) in NCBI with an E-value cut-off of 1.0 e-5.

### Identification OBP of and CSP genes

Putative TeleOBP and TeleCSP genes were identified by searching odorant binding protein and chemosensory protein keywords in the annotated unigenes, and by using BLAST. Annotated OBP and CSP genes from other Hemipteran species, such as *A*. *suturalis* Jakovlev, *A*. *lineolatus* Goeze, *A*. *lucorum* Meyer-Dür, *N*. *ericae* Schilling, *Halyomorpha halys* Stål, and *L*. *lineolaris* Palisot de Beauvois were used as references. The putative *T*. *elegans* OBP and CSP genes were confirmed by searching against the NCBI non-redundant (nr) protein database using BlastX with cut-off *E*-value of 10–5.

### Bioinformatic analysis

Signal peptides were predicted using SignalP 4.1 server^47^ (http://www.cbs.dtu.dk/services/SignalP/). The similarity searches were performed using NCBI BLAST (http://blast.ncbi.nlm.nih.gov/). Multiple sequence alignment was conducted using DNAMAN 6.0. Amino acid sequence alignments of the matured OBPs and CSPs from *T*. *elegans* and other Hemipteran species were performed using MAFFT (http://mafft.cbrc.jp/alignment/server/clustering.html), and phylogenetic trees were constructed using PhyML^48^ in Seaview v.4 using the Jones-Taylor-Thomton (JTT) model with 1000-fold bootstrap replication in neighbor-joining method (NJ).

### Tissue specific expression of OBPs and CSPs

Antennae (300 pairs), heads (without antennae) (180), thoraxes (120), abdomens (50), legs (300 pairs) and wings (200 pairs) of male and female adults at 3-day after eclosion were excised and immediately frozen in liquid nitrogen. All total RNA samples were extracted using the RNAiso Plus kit (TaKaRa, Dalian, China) and the isolated RNA was transcribed to first-strand cDNA by PrimeScript^TM^ RT reagent Kit with gDNA Eraser (TaKaRa, Dalian, China) following the manufacturer’s instructions. The nucleotide sequences of all 19 TeleOBPs and 7 TeleCSPs were confirmed by cloning and sequencing (Figure S1). Real-time quantitative PCRs (RT-qPCRs) were performed with SYBR^®^ Premix Ex Taq^TM^ II (TaKaRa, Dalian, China). The *T*. *elegans* actin gene (Genbank accession no. MG322127) was used as control. Each reaction was performed with 200 ng/μl cDNA sample. Primers for RT-qPCR were designed using Primer Premier 5.0 software and are listed in Table S2. The RT-qPCR reactions were conducted in 20 μL reaction mixtures containing 10 μL SYBR Premix Ex Taq II, 20 ng cDNA templates, 0.2 μM of each primer, and nuclease-free water. The cycling conditions were: one cycle of 95 °C for 5 min, followed by 40 cycles of 95 °C for 5 s and 55 °C for 30 s. Melt curve conditions were 95 °C for 10 s, 65 °C for 30 s. A no-template control (NTC) was also included to detect possible contamination. Three biological replicates were analysed and relative expression levels of OBP and CSP genes across the samples were measured using the 2^−∆∆*C*T^ method^49^. The differences in the expression of TeleOBP and TeleCSP genes between female and male tissues were compared by a one-way nested analysis of variance (ANOVA), followed by Tukey’s honestly significance difference (HSD) test using SPSS (SPSS Institute 17.0, IBM, Chicago, IL, USA).

## Electronic supplementary material

Supplementary Information
