# Supplementary material for: Identification and tissue distribution of chemosensory protein and odorant binding protein genes in Tropidothorax elegans Distant (Hemiptera: Lygaeidae)
Source: Sci Rep. 2018 May 17;8:7803. doi: 10.1038/s41598-018-26137-6 (PMC5958050; doi:10.1038/s41598-018-26137-6)
Supplement: Supplementary file 1 — Supplementary Information [file 41598_2018_26137_MOESM1_ESM.pdf]

Supplementary

Identification and tissue distribution of chemosensory protein and odorant binding protein genes in *Tropidothorax elegans* Distant (Hemiptera: Lygaeidae)

Yue-Qin Song[1], Hui-Zhong Sun[1]& Jun Du[2]

[1]Forestry College, Henan University of Science and Technology, Luoyang 471000, China

[2]Institute of Plant Nutrition and Resource Environment, Henan Academy of Agricultural Sciences, Zhengzhou 450002, China

Supplementary Table 1 Assembly quality of *Yemma signatus* antennal transcriptome

Sequence length (bp)	Transcripts		Unigenes	
	Number	Percentage (%)	Number	Percentage (%)
200~300	39 028	32.80	37 292	39.70
300~500	26 978	22.67	23 569	25.09
500~1 000	24 175	20.32	17 834	18.98
1 000~2 000	16 673	14.01	9 419	10.03
>2 000	12 144	10.21	5 826	6.20
Total number	118 998		93 940	
Total length	101 486 213		62 690 178	
N50 length (bp)	1 545		1 046	
Mean length (bp)	852.84		667.34	

Supplementary Table 2 Primers used for gene cloning and sequencing

Primer name	Sequence (5'-3')	Primer name	Sequence (5'-3')
TeleOBP1-F	ATGTACCAGCTACTGCCTC	TeleOBP14-F	ATGAACGGACACATCTTC
TeleOBP1-R	AGAGGCCTCCTCCATTTTAG	TeleOBP14-R	TCAGGGATTGAAGAAATCA
TeleOBP2-F	AGTGGAGCCAGCAAAGAG	TeleOBP15-F	ATGAAGGTGCTCCTCGTAC
TeleOBP2-R	TTACGGCACGATGAATGC	TeleOBP15-R	TCACCATAGTTCTGAACT
TeleOBP3-F	ATGGCCGCTCAAAGAACC	TeleOBP16-F	ATGAAGTACACTGTATC
TeleOBP3-R	TCATGTAGGGGGCTTCACCCCTTT	TeleOBP16-R	TTACTCTTTGGCAGCTT
TeleOBP4-F	ATGTACTTCCTTGCACTA	TeleOBP17-F	ATGGCTCCCACCTACGT
TeleOBP4-R	CTTCTTCAAGGTAACACTTG	TeleOBP17-R	TTAGATCATTCCAACCTC
TeleOBP5-F	ATGCTGCGCCACTTCTCGC	TeleOBP18-F	ATGAAAGCGTATTCCATT
TeleOBP5-R	TTATCCTCTACTCTGAAA	TeleOBP18-R	TCAGTGGTCATGTTTTTTCAATGGTTG
TeleOBP6-F	AGTGCTCAACAAGGTAGT	TeleOBP19-F	ATGAGGTCAGTGTTTGTAG
TeleOBP6-R	GATATTTTCTGAGTTTCTTTCGTCGC	TeleOBP19-R	CTAGTTTCTTGGTTTTGGAGG
TeleOBP7-F	ATGTCCCCAAGCAAGCTT	TeleCSP1-F	ATGAAGTGCCTACTCGCA
TeleOBP7-R	TCATATTCCAAAGCGTTC	TeleCSP1-R	TTAGCTCGGCAATTTGAT
TeleOBP8-F	ATGTTGACCAACTTCATC	TeleCSP2-F	CAGGCAGTACGTCACCAGT
TeleOBP8-R	TCACATTTTGGAATTGACACCC	TeleCSP2-R	TTAGCCCAAGTATTTGGA
TeleOBP9-F	ATGGAAGTGATTGTGATGAG	TeleCSP3-F	ATGAAGATCATCATTGCTC
TeleOBP9-R	TTACTTATTCCCGAAACA	TeleCSP3-R	TCAAACGGCGATTCCTT
TeleOBP10-F	ACCAGGCCTCCAACCTGCAT	TeleCSP4-F	ATGCTCAAGATAAGAACT
TeleOBP10-R	TCAGTGGTTGAATTGAGGT	TeleCSP4-R	CTAGTCCTCATAGGTAGGT
TeleOBP11-F	ATGACTTCCTTCAGATCT	TeleCSP5-F	ATGCAAATCTCAGGAGTC
TeleOBP11-R	TCAATGTTTTTCTTCGTGTATC	TeleCSP5-R	TTATCCCCTGGGAGTGTC
TeleOBP12-F	ATGATGATCTCATGTAAT	TeleCSP6-F	ATGAAGCTGGCCCTAGTAGT
TeleOBP12-R	TTAGCTCTTCAACTTCTT	TeleCSP6-R	TCAAGAAGACTGGACGAG
TeleOBP13-F	ATGCTCCTAGCTCTCAGT	TeleCSP7-F	ATGTCTCTCTTCGGATCCG
TeleOBP13-R	TCACTGAGATTCACAGT	TeleCSP7-R	TTAATATGTGGGGGGTGGTTT

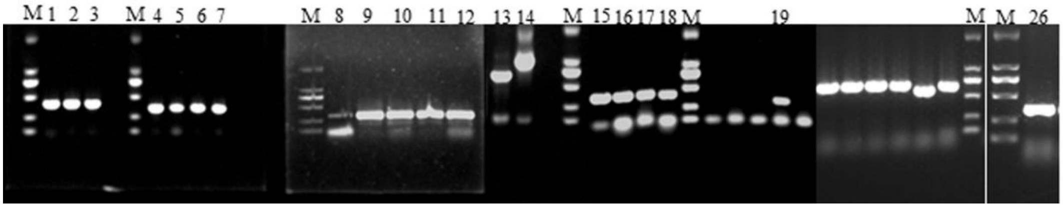

Supplementary Figure 1 The agarose gel electrophoresis diagram of gene cloning

M, D2000 marker; 1, TeleOBP3; 2, TeleOBP14; 3, TeleOBP16; 4, TeleOBP4; 5, TeleOBP17; 6, TeleOBP10; 7, TeleCSP4; 8, TeleOBP12; 9, TeleCSP2; 10, TeleCSP3; 11, TeleCSP5; 12, TeleCSP6; 13, TeleOBP18; 14, TeleOBP5; 15, TeleCSP7; 16, TeleOBP15; 17, TeleOBP11; 18, TeleOBP6; 19, TeleCSP1; 20, TeleOBP13; 21, TeleOBP7; 22, TeleOBP8; 23, TeleOBP1; 24, TeleOBP9; 25, TeleOBP19; 26, TeleOBP2.
